# Protective Effect of Adenosine A_2B_ Receptor Agonist, BAY60-6583, Against Transient Focal Brain Ischemia in Rat

**DOI:** 10.3389/fphar.2020.588757

**Published:** 2021-02-11

**Authors:** Ilaria Dettori, Lisa Gaviano, Filippo Ugolini, Daniele Lana, Irene Bulli, Giada Magni, Francesca Rossi, Maria Grazia Giovannini, Felicita Pedata

**Affiliations:** ^1^ Department of Neuroscience, Psychology, Drug Research and Child Health (NEUROFARBA), Division of Pharmacology and Toxicology, University of Florence, Florence, Italy; ^2^ Department of Health Sciences, Section of Clinical Pharmacology and Oncology, University of Florence, Florence, Italy; ^3^ Institute of Applied Physics “Nello Carrara”, National Research Council (IFAC-CNR), Florence, Italy

**Keywords:** adenosine A_2B_ agonist, cerebral ischemia, middle cerebral artery occlusion, neuroinflammation, blood biomarkers

## Abstract

Cerebral ischemia is a multifactorial pathology characterized first by an acute injury, due to excitotoxicity, followed by a secondary brain injury that develops hours to days after ischemia. During ischemia, adenosine acts as an endogenous neuroprotectant. Few studies have investigated the role of A_2B_ receptor in brain ischemia because of the low potency of adenosine for it and the few selective ligands developed so far. A_2B_ receptors are scarcely but widely distributed in the brain on neurons, glial and endothelial cells and on hematopoietic cells, lymphocytes and neutrophils, where they exert mainly anti-inflammatory effects, inhibiting vascular adhesion and inflammatory cells migration. Aim of this work was to verify whether chronic administration of the A_2B_ agonist, BAY60-6583 (0.1 mg/kg i.p., twice/day), starting 4 h after focal ischemia induced by transient (1 h) Middle Cerebral Artery occlusion (tMCAo) in the rat, was protective after the ischemic insult. BAY60-6583 improved the neurological deficit up to 7 days after tMCAo. Seven days after ischemia BAY60-6583 reduced significantly the ischemic brain damage in cortex and striatum, counteracted ischemia-induced neuronal death, reduced microglia activation and astrocytes alteration. Moreover, it decreased the expression of TNF-α and increased that of IL-10 in peripheral plasma. Two days after ischemia BAY60-6583 reduced blood cell infiltration in the ischemic cortex. The present study indicates that A_2B_ receptors stimulation can attenuate the neuroinflammation that develops after ischemia, suggesting that A_2B_ receptors may represent a new interesting pharmacological target to protect from degeneration after brain ischemia.

## Introduction

Stroke is the second leading cause of death (Mozaffarian et al., 2015) and the third leading cause of disability (Murray et al., 2012) in Western Countries and is considered a disease of immense importance for public health, with serious economic and social consequences. The accepted pharmacological strategy consists of tissue-type plasminogen activator (tPA) administration (Fonarow et al., 2011) within the first 4–4.5 h after ischemia.

The role of adenosine receptors has been deeply investigated during ischemia (Ongini et al., 1997; Cunha, 2001; Ribeiro et al., 2002; Schwarzschild et al., 2002; Fredholm and Svenningsson, 2003; Chen et al., 2007; Pedata et al., 2016). However, so far only few studies have explored the role of A_2B_ receptors in brain ischemia. Indeed, the A_2B_ receptor subtype is the least studied among adenosine receptors and still remains the most enigmatic subtype because of the relatively low potency of adenosine for it (EC50 24 μM) (Fredholm et al., 2011), and the limited availability of selective agonists so far described.

It is well known that the acute pathogenic mechanisms after the ischemic insult consists of excitotoxicity and peri-infarct depolarization and that a few hours after the onset of ischemia, activation of microglia, the resident immune cells, and production or activation of inflammatory mediators take place (Dirnagl et al., 1999). Protracted neuroinflammation is now recognized as the predominant mechanism of secondary brain injury progression, being characterized by damage to the endothelium and to the blood brain barrier (BBB) and infiltration of peripheral leukocytes (neutrophils, lymphocytes and monocytes) and macrophages in brain parenchyma, that contribute to expand the ischemic damage. Thus, besides the approved treatment with tPA in the first hours after ischemia, an important strategy to counteract the ischemic damage is to control brain injury progression after ischemia. Studies in mice ablated of A_2B_ receptors on bone marrow cells indicate an important contribution of vascular A_2B_ receptors in attenuating vascular leakage during hypoxia (Eckle et al., 2008). Indeed, it was found that activation of A_2B_ receptors in a model of femoral artery injury is vasoprotective (Yang et al., 2006; Chen et al., 2011).

In addition to brain cells and endothelial cells, A_2B_ receptors are present on hematopoietic cells, such as lymphocytes and neutrophils, with the highest level of expression on macrophages (Gessi et al., 2005; Yang et al., 2006; Eckle et al., 2008). A_2B_ receptors in most cases are coexpressed with A_2A_ receptors on hematopoietic cells and their activation exerts anti-inflammatory effects, inhibiting vascular adhesion (Yang et al., 2006) and migration of inflammatory cells (Wakai et al., 2001; Konrad et al., 2012). Thus, attenuation of hypoxia-associated increases of tissue neutrophils due to infiltration in different tissues including the brain, may largely depend on hematopoietic cell A_2B_ signaling (Eckle et al., 2008).

We recently reported that, 7 days after transient Middle Cerebral Artery occlusion (tMCAo), a time when intense inflammatory reaction is still ongoing, pro-inflammatory cytokines such as Interleukin-1β (IL-1β) and Tumor Necrosis Factor-α (TNF-α) are significantly increased in peripheral blood, while the antinflammatory cytokine Interleukin-10 (IL-10) is decreased (Dettori et al., 2018). Significant increases of TNF-α serum levels are also found in patients with acute stroke, reaching peak values on day 7 (Intiso et al., 2004). Thus, TNF-α increase and IL-10 decrease may represent valuable blood markers of the brain damage following an ischemic insult (Jickling and Sharp, 2011).

Aim of the present work was to verify whether chronic administration of the A_2B_ receptor agonist BAY60-6583, starting 4 h after focal ischemia caused by tMCAo in the rat, has protective effects on the ischemic damage and on the neurological score evaluated 7 days after the ischemic insult. We studied the protective effect, if any, of BAY60-6583 on neurodegeneration, astrocytes and microglia activation and on mediators of inflammation in the peripheral blood. Moreover, we investigated if BAY60-6583 prevented leukocyte infiltration into brain parenchyma.

## Materials and Methods

### Animals

Male Wistar rats (Envigo, Italy) weighting 270–290 g were used. Animals were housed in groups of three with free access to food and water and kept under standardized temperature, humidity and light conditions (12 h light/dark cycle) in the animal house facility of the University of Florence. The experimental procedures were conducted in accordance with the ARRIVE guidelines and were authorized by the Italian Ministry of Health. The ethical policy of the University of Florence complies with to the Directive 2010/63/EU of the European Parliament and to the Italian Regulation DL 26/2014 on the protection of animals used for scientific purposes. According to the law, all efforts were made to fulfill to the principle of 3 Rs.

### Surgery

Focal cerebral ischemia was induced in the right hemisphere by intraluminal tMCAo. The animals were anesthetized with 5.0% isoflurane (Baxter International) and spontaneously inhaled 1.0–2.0% isoflurane in air with the aid of a mask. Body core temperature was maintained at 37°C with a recirculating pad and K module and was monitored via an intrarectal type T thermocouple (Harvard, Kent, United Kingdom). The surgical procedure to occlude the MCA consisted in the insertion of a 4–0 nylon monofilament (Doccol corporation, Sharon, MA, United States), via the external carotid artery into the internal carotid artery in order to block the origin of the MCA, according to the procedure described by (Melani et al., 1999). One hour after occlusion, animals were re-anesthetized with isoflurane, the filament was withdrawn, thus allowing the reperfusion of the brain. The sham operation was carried out inserting the filament into the internal carotid artery and immediately withdrawing it. Carprofen (5 mg/kg) was administered intraperitoneally (i.p.) to reduce post-operative pain. To confirm MCAo, we evaluated the “intra-ischemic score” and the presence of circling behavior (parameters evaluated inside the 1 h period of MCAo after rat awakening from anesthesia). The “intra-ischemic score” was calculated by evaluation of: 1) the palpebral fissure with an ellipsoidal shape; 2) laterally extension of one or both ears; 3) asymmetric body bending on the ischemic side; 4) limb laterally extension and not alignment to the body. The circling behavior is an acute behavioral response not anymore shown 24 h after MCAo (Hunter et al., 2000; Melani et al., 2003).

### Inclusion Criteria

Circling behavior after awakening from anesthesia during MCAo.

Intra-ischemic score >3.

### Exclusion Criteria

No ischemic lesion at histology.

Major protocol violation (i.e., errors in ischemia time).

### Drug Administration and Experimental Groups

The adenosine A_2B_ receptor agonist 2-[[6-Amino-3,5-dicyano-4-[4-(cyclopropylmethoxy) phenyl]-2-pyridinyl] thio]-acetamide (BAY60-6583) (Tocris, Bristol, United Kingdom), was dissolved in dimethyl sulfoxide (DMSO) 0.5% in saline and was administered i.p. at the dose of 0.1 mg/kg (800 µl), twice per day for 7 days starting 4 h after occlusion. The administration protocol used was the same as that already described (Melani et al., 2014), that demonstrated the protective effect of the adenosine A_2A_ receptor agonist CGS21680 in the tMCAo model in the rat. The dose of BAY60-6583 (0.1 mg/kg) was chosen by comparison with the protective dose of CGS21680 (Melani et al., 2014), taking into account the affinity of BAY60-6583 and CGS21680 for the rat A_2B_ and A_2A_ receptors, respectively (Alnouri et al., 2015). A further criterion was to refer to the dose of BAY60–6583 (0.1 mg/kg) which in previous studies had shown protection from peripheral arterial lesions (Bot et al., 2012) and from lung injury induced by mechanical ventilation in mice (Eckle et al., 2008).

A total of 38 rats were operated, six animals died 24 h after operation: one BAY60–6583-treated rat and five vehicle-treated rats. Three animals were excluded: one for drug administration protocol violation and two because they did not show any infarct area at the histological analysis.

A group of animals was operated as described above and sacrificed 7 days after tMCAo. Rats were randomly allocated to the following groups: 1) sham-operated (sham) rats (n = 7) that did not receive any treatment; 2) tMCAo + vehicle-treated rats (n = 6) treated i.p. with 800 µl of 0.5% DMSO in saline (vehicle), administered twice per day for 7 days, starting 4 h after tMCAo; 3) tMCAo + BAY60-6583-treated rats (n = 6) treated with BAY60-6583 dissolved in vehicle and administered i.p. twice per day for 7 days, starting 4 h after tMCAo.

A second group of animals was operated as described above and sacrificed 2 days after tMCAo. Rats were randomly allocated to the following groups treated as described above: 1) sham rats (n = 3); 2) tMCAo + vehicle-treated rats (n = 3); 3) tMCAo + BAY60-6583-treated rats (n = 4).

### Neurological Deficit

The neurological deficit was evaluated by the modified Neurological Severity Score (mNSS) test described by (Chen et al., 2001). The examiners were blind both to the type of surgery and to the treatment. All tests were carried out before tMCAo and 1, 5 and 7 days after tMCAo. The mNSS test evaluates the sensorimotor deficit: it is composed of motor, sensory, reflex and beam balance tests. The score assigned to each rat at completion of the evaluation equals the sum of all test scores. The test is graded on a scale from 0 (normal score) to 18 (maximal deficit score). In the beam balance test, a score between 0 (normal score) and 6 (maximal deficit score) was assigned to each animal in function of the ability to stay and walk on the beam. Beam balance test score affects 1/3 of the total mNSS score.

### Body Weight

The time course of the rats’ body weight was evaluated before tMCAo and 1, 5, 7 days after artery occlusion. The body weight variation after ischemia was expressed as the difference in g of each rat’s body weight minus its own pre-operation body weight.

### Ischemic Brain Damage

At day 7, rats were anesthetized with Zoletil 50/50 (100 mg/kg i.p., Virbac, Carros, Francia) and were perfused transcardially with 500 ml of an ice-cold 4% paraformaldehyde in phosphate buffer solution (pH 7.4). The brains were collected, post-fixed overnight and cryoprotected in cold 18% sucrose solution in phosphate buffer for at least 48 h. Coronal sections (30 µm) were cut with a cryostat, were collected at 210 µm intervals at 12 different levels through the striatum (AP: from +2.2 to −2.0 mm from Bregma) (König and Klippel, 1967), collecting three adjacent sections at each level. Then coronal sections were stored at −20°C in anti-freeze solution until staining. Coronal brain sections from the groups at 7 days after tMCAo were stained using acetate cresyl violet (1%) or hematoxylin and eosin (H&E). Histological analysis with cresyl violet staining allows to clearly define the infarct area and volume up to 1 week after ischemia (Rousselet et al., 2012). To evaluate the area and volume of the ischemic damage, 12 cresyl violet-stained brain sections/rat were placed directly on the scanning screen of a color flatbed scanner (CanoScan LiDE 90; Canon). Following image acquisition, the images were analyzed using ImageJ software. The measurements of the infarct area in striatum and cortex were obtained outlining manually the margins of the infarcted areas. Ischemic cortical and striatal volumes were calculated multiplying the infarcted area by the section thickness and summing the volume of the 12 sections. After H&E staining, heterochromatic nuclei were counted at Bregma level within an optical field at 20X in ischemic cortex and striatum. Quantitative analysis was conducted blind to the treatment group and data were then averaged and expressed as mean ± SEM of number cells per optical field of “n” animals.

### Neuronal Damage, Gliosis and Blood Cell Infiltration

Immunohistochemistry was performed on 30 µm-thick brain coronal sections of striatum and cortex (AP: between 0 and +0.2 mm from Bregma) with the free-floating method (Giovannini, 2002; Lana et al., 2014).

The following primary antibodies were used. For neurons, a mouse anti-neuronal nuclei (NeuN) antibody (1:400, Product code: #MAB377, Millipore, Billerica, MA, United States); for astrocytes, a mouse anti-Glial Fibrillary Acidic Protein (GFAP) antibody conjugated with the fluorochrome AlexaFluor 488 (1:500, Product code: #MAB3402X, Millipore, Billerica, MA, United States); for microglia, a rabbit anti-ionized calcium binding adaptor molecule 1 (IBA1) antibody (1:400, Product code: #016-20001, WAKO, Osaka, Japan); for granulocytes, a mouse monoclonal antibody anti-HIS-48 (1:50, Product code: #sc-19613, Santa Cruz Biotechnology, Heidelberg, Germany). Cytochrome C (CytC) was stained using a mouse monoclonal anti-CytC (1:200, Product code: #556432, BD Pharmingen, San Jose, CA, United States). The following fluorescent secondary antibodies were used: AlexaFluor 555 donkey anti-mouse (1:400, Product code: #A31570, Thermo Fisher Scientific, Altrincham, United Kingdom); goat anti-mouse IgG-FITC (1:400, Product code: #sc-2094, Santa Cruz Biotechnology, Heidelberg, Germany); AlexaFluor 635 goat anti-rabbit (1:400, Product code: #A31577, Thermo Fisher Scientific, Altrincham, United Kingdom). Nuclei were stained using DAPI, contained in the mounting medium for glass slides, Vectashield (Vector Laboratories, Burlingame, CA, United States).

Procedure for the free-floating immunostaining:

Day 1. Striatal sections (thickness 30 µm) were placed in wells of 24-well plates and were rinsed three times (5 min each) in PBS-B-TX (PBS-B-0.3% Triton X-100) and blocked for 60 min with 500 µl Blocking Buffer (BB) (10% normal goat serum in PBS-TX and 0.05% NaN_3_). Sections were incubated overnight at 4°C under slight agitation with a combination of two primary antibodies, as necessary, both dissolved in BB.

Day 2. For triple immunostaining, after washings (three times, 5 min each with PBS-B-TX), the sections were incubated for 90 min at room temperature in the dark with AlexaFluor 555 donkey anti-mouse IgG (1:400) secondary antibody diluted in BB. Then for 2 h at room temperature in the dark with AlexaFluor 555 donkey anti-mouse IgG (1:400) plus AlexaFluor 635 goat anti-rabbit IgG (1:400). For triple immunostaining, after washings, astrocytes were immunostained using a mouse anti-GFAP primary antibody directly conjugated with the fluorochrome AlexaFluor 488 (1:500).

After extensive washings, the sections were mounted onto gelatin-coated slides using Vectashield with DAPI (Vector Laboratories). Slices were observed with LEICA TCS SP5 a confocal laser scanning microscope (Leica Microsystems CMS GmbH, Manheim, Germany) and with an epifluorescent Olympus BX63 microscope (Olympus, Hamburg, Germany) and photographed using a digital camera (Olympus DP50).

### Microscopy Techniques, Qualitative and Quantitative Analysis

The confocal microscopy images were obtained with a LEICA TCS SP5 confocal laser scanning microscope. The parameters of acquisition were maintained constant: frame dimension 1,024 × 1,024 points, frequency of acquisition 200 Hz. Quantitative analyses of neurons, astrocytes and microglia were performed in a striatal area within the peri-infarctual region or boundary zone (region of interest, ROI), where the insult is less intense, and neurons are still salvageable. Three confocal acquisition were taken in the ROI, laterally to the Lateral Ventricle (L between 1.5 and 2.2 mm from Bregma in the right hemisphere), in a dorsoventral sequence (H between −4 and −6.1 mm from the surface), and data collected from the three confocal scans were averaged. All quantitative analyses were performed using ImageJ software (National Institute of Health).

All quantitative analyses described here below were performed blind to the treatment by two independent experimenters and data were averaged. All evaluations of cell density were made on z stacks of 10 consecutive confocal scans (total thickness 12 µm). Cells were counted in the ROI, the area of analyses was measured and cell numbers were expressed as density (number of cells/mm^2^). LDN neurons (Fusco et al., 2018), quantified as LDN neurons/mm^2^ were expressed as percent of total neurons in the same area. The immunofluorescence of GFAP or IBA1 per cell was calculated in pixel over a set threshold and divided for the total number of cells in the same ROI. The number of pixel is proportional to the cell volume.

To evaluate the number of granulocytes, HIS-48^+^ cells were counted at seven different levels per animal (AP: +2.0 to −1.0 mm from the Bregma) within an optical field at 20X in ischemic cortex and striatum. Quantitative analyses were then averaged and expressed as mean ± SEM of number cells per optical field of “n” animals.

### Determination of TNF-α and IL-10 in the Spleen

Seven days after tMCAo the spleens were collected and smashed by mechanical dissociation on nylon filters with 70 μm pores (Corning), followed by Ficoll-Paque density gradient centrifugation assay to obtain plasma samples. The levels of the pro-inflammatory cytokine TNF-α and of the regulatory cytokine IL-10 were measured on aliquots (100 µl) of plasma using commercial ELISA kits (Rat TNF-α Platinum ELISA, Catalog no: BMS622, Affymetrix eBioscience, Vienna, Austria; Rat IL-10 Platinum ELISA, Catalog no: BMS629, Affymetrix eBioscience, Vienna, Austria), following the protocol provided by the manufacturer. Results were expressed as pg protein/ml plasma.

### Statistical Analysis

Data were statistically analyzed by one-way analyses of variance (ANOVA) followed by Newman-Keuls multiple comparison test, two-way analyses of variance (ANOVA) followed by Bonferroni post hoc test or Repeated Measures two-way analyses of variance (ANOVA) followed by Tukey post hoc test, or by unpaired Student’s t test, as appropriate. A value of at least *p* < 0.05 was considered statistically significant. The statistical analyses were performed utilizing GraphPad Prism7.

## Results

### Effect of Treatment With the Adenosine A_2B_ Receptor Agonist on Neurological Deficit and on Body Weight Loss After tMCAo

**FIGURE 1:**
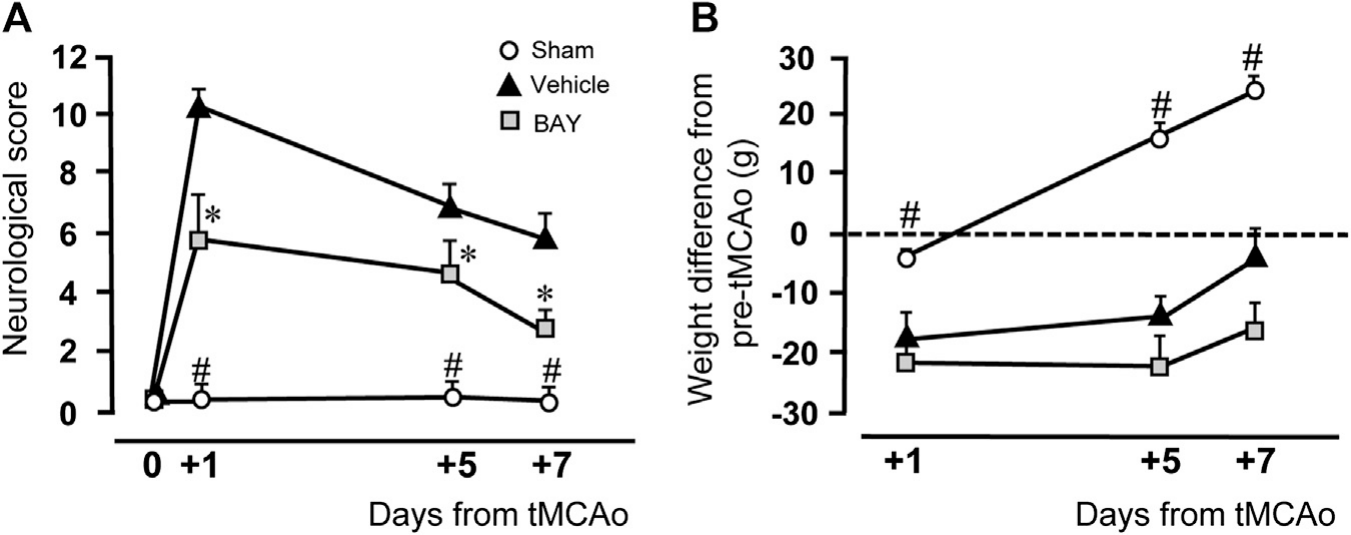
Effect of BAY60**-**6583 (0.1 mg/kg, i.p.) on neurological deficit and body weight. **(A)** The score of mNSS test was evaluated before and 1, 5 and 7 days after tMCAo. Statistical significance was evaluated by Repeated Measures two-way ANOVA followed by Tukey post hoc test. Significativity: ^#^at least *p* < 0.03, sham vs. BAY60-6583- and vehicle-treated rats; *at least *p* < 0.05, BAY60-6583- vs. vehicle-treated rats. **(B)** The body weight was calculated as the difference in g of the rat’s body weight at each time point minus its own pre-operation body weight. Significativity: ^#^at least *p* < 0.03, sham vs. BAY60-6583- and vehicle-treated rats, Repeated Measures two-way ANOVA followed by Tukey post hoc test.

Figure 1A shows the neurological score evaluated by the mNSS test before and 1, 5, and 7 days after ischemia. Sham rats had no neurological deficit, as shown by the neurological score of 0.1–0.6 from day 0 to day 7 after tMCAo. At day 1 after tMCAo, vehicle-treated rats had a clear neurological deficit, shown by the neurological score of 10.3 ± 0.9, which defines a moderate injury. The neurological impairment recovered spontaneously, although not completely, at 5 and 7 days after tMCAo. As shown in Figure 1A, the neurological score was reduced 5 days after tMCAo to 6.7 ± 0.6, and 7 days after tMCAo to 5.8 ± 0.9. Treatment with BAY60-6583 (0.1 mg/kg) significantly reduced the neurological deficit at each time point after tMCAo. Repeated Measures two-way ANOVA calculated for the two factors: Treatment and Time after tMCAo, showed that Treatment (F_2,16_ = 37.2; *p* < 0.0001), Time (F_2,32_ = 17.0; *p* < 0.0001) and Interaction between Treatment and Time (F_4,32_ = 3.9; *p* < 0.009) were statistically significant. The Tukey post hoc test indicated that vehicle- and BAY60-6583-treated rats had a neurological score significantly different from both sham rats at each time point (1, 5, 7 days after tMCAo, *at least *p* < 0.03). Treatment with BAY60-6583 significantly reduced the neurological deficit in comparison to vehicle-treated rats at 1, 5 and 7 days (*at least *p* < 0.02) after tMCAo.

Figure 1B shows the time course of the body weight variations after tMCAo, expressed as difference from the rat’s body weight determined after and before tMCAo. Sham rats had a slight, not significant, body weight loss 1 day after tMCAo. Their body weight increased steadily and significantly at day 5 (+16.4 ± 2.2 g) and day 7 (+26.6 ± 2.1 g) after tMCAo. The body weight of vehicle-treated rats decreased significantly 1 day after tMCAo (−17.60 ± 4.5 g), and, although it increased steadily at day 5 (−14.0 ± 3.6 g) and day 7 (−0.6 ± 4.1 g) after tMCAo, it was always lower than sham rats. The A_2B_ receptor agonist, BAY60-6583, had no effect on the recovery of body weight loss at any time point after tMCAo, as compared to vehicle-treated rats. Statistical analysis performed with Repeated Measures two-way ANOVA, calculated for the two factors: Treatment and Time after tMCAo, showed that Treatment (F_2,15_ = 29.7; *p* < 0.0001), Time (F_2,30_ = 68.5; *p* < 0.0001) and the Interaction between Treatment and Time (F_4,30_ = 13.7; *p* < 0.0001) were statistically significant. The Tukey post hoc test indicated that the body weight of sham rats was significantly higher than that of both vehicle-treated and BAY60-6583-treated rats at days 1, 5, and 7 after tMCAo (*at least *p* < 0.03). Treatment with BAY60-6583 did not modify significantly the body weight loss in comparison to vehicle-treated rats at all time points.

### Effect of Treatment With the Adenosine A_2B_ Receptor Agonist on Brain Ischemic Damage After tMCAo

**FIGURE 2:**
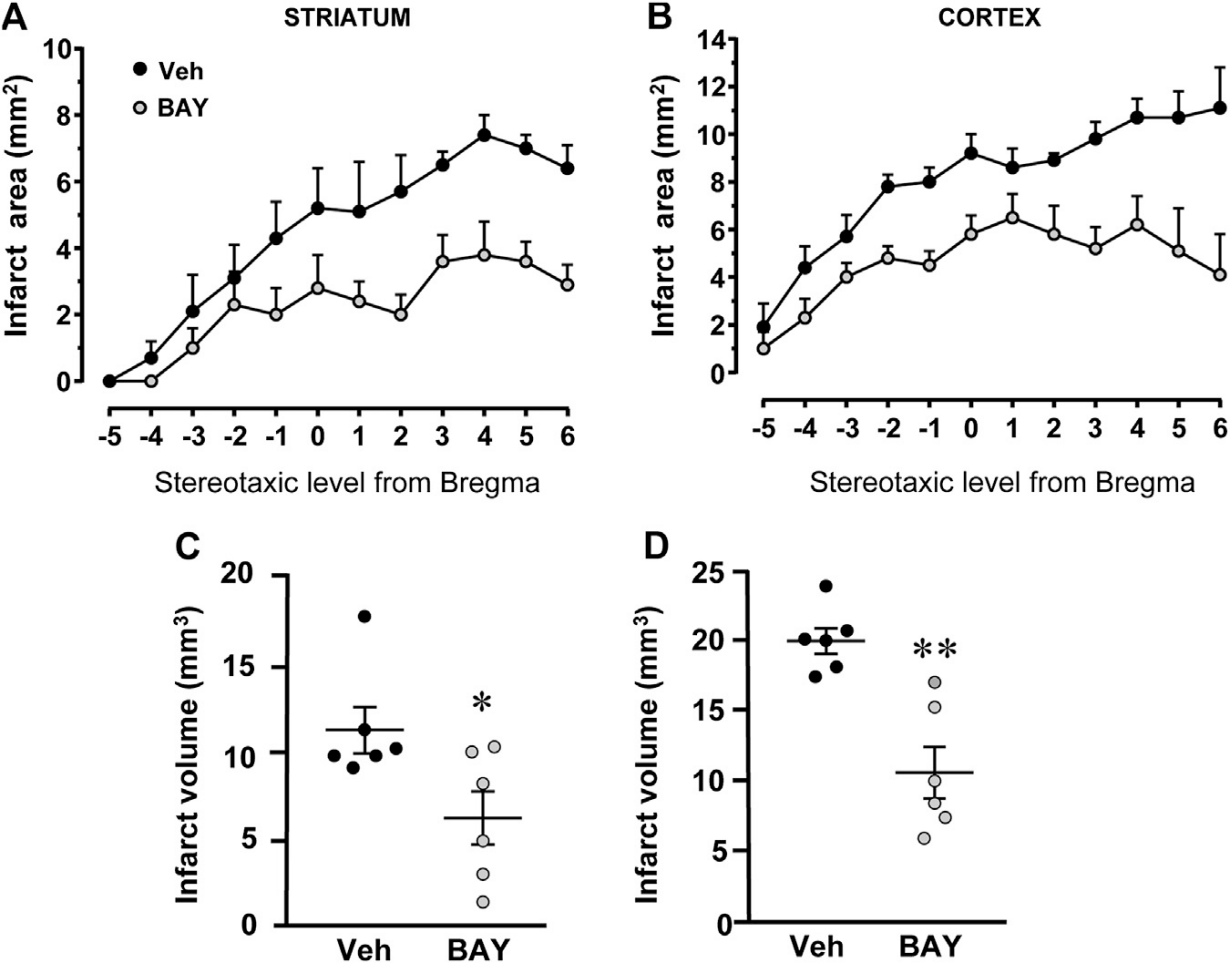
Effect of chronic treatment with BAY60**-**6583 (0.1 mg/kg i.p.) on infarct area and infarct volume in the striatum and cortex 7 days after tMCAo. **(A,B)** Data represent infarct area measured at 12 coronal levels between AP: +2.2 mm to −2.0 mm from Bregma of vehicle- (n = 6) and BAY60-6583-treated rats (n = 6) (König et al., 1964). **(C,D)** Graphs show the infarct volume calculated in the striatum and cortex as mean ± SEM (individual data points are shown) of n = 6 rats per group. Significativity: *p < 0.05; **p < 0.001 vs. vehicle-treated rats, unpaired Student’s t-test.

Figure 2 shows the extent of the ischemic damage evaluated as infarct area (Figures 2A,B) and infarct volume (Figures 2C,D) in the striatum and cortex of vehicle- and BAY60-6583-treated rats 7 days after tMCAo. Chronic treatment with BAY60-6583, at the dose of 0.1 mg/kg, significantly reduced the infarct area and volume in both areas. In BAY60-6583-treated rats the striatal and cortical infarct volumes were reduced by 44.7% and 46.3%, respectively (*p < 0.05, **p < 0.001 vs. vehicle-treated rats, unpaired Student’s t-test: Figures 2C,D). In BAY60-6583-treated rats the striatal and cortical infarct volumes were reduced by 44.7% and 46.3%, respectively (*p < 0.05, **p < 0.001 vs. vehicle-treated rats, unpaired Student’s t-test: Figures 2C,D). Sham rats did not show any damage both in the striatum and in the cortex (not shown).

**FIGURE 3:**
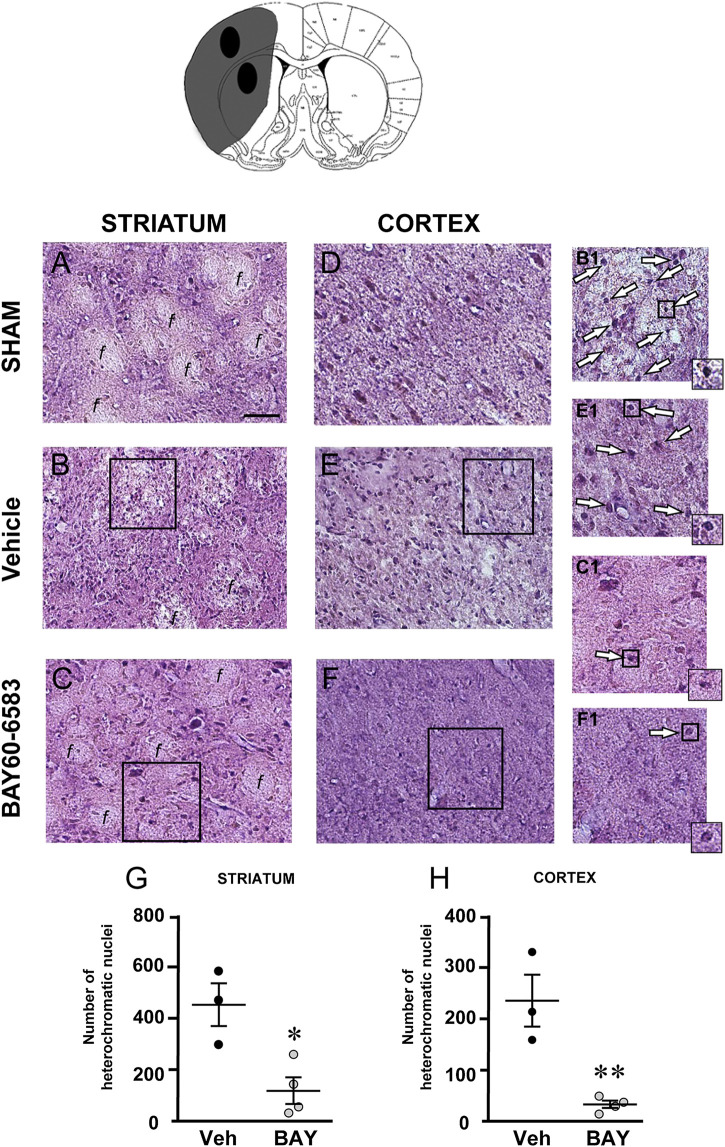
Effect of BAY60**-**6583 (0.1 mg/kg, i.p.) on the cytoarchitecture of the striatum and cortex 7 days after tMCAo. Upper part: Schematic brain picture of a coronal section (at Bregma = 0, König et al., 1964) showing the infarct area in a vehicle-treated rat. The two black circles indicate regions within the infarct area where photomicrographs were captured. **(A–F)** Representative microphotographs of H&E staining from dorsal striatum and fronto-parietal cortex of a sham **(A,D)**, a vehicle- **(B,E)** and a BAY60-6583-treated rat **(C,F)**. The white matter *fascicula* (*f*) are evidenced. Scale bar = 100 µm. Insets show magnifications of cells present in the square areas of panels **(C–F)**. **(G,H)** Quantitative analyses of heterochromatic nuclei per striatal **(G)** and cortical areas **(H)** at coronal level AP = 0 from Bregma. Data represent the mean ± SEM (individual data points are shown) of three rats/group (*p < 0.02; **p < 0.005 BAY60-6583- vs. vehicle-treated rats, unpaired Student’s t test.

To characterize the cytoarchitecture of the ischemic cortex and striatum at day 7 after tMCAo, striatal and cortical sections from the three experimental groups were stained with H&E (Figures 3A–F). Seven days after transient ischemia, H&E staining showed a decrease of staining intensity in vehicle-treated rats (Figures 3B,E) compared to the sham rats (Figures 3A,D). The decrease of staining intensity is attributable to an increase in extracellular fluid consequent to oedema. The typical cytoarchitecture of cortex and striatum was lost (for a description see (Danner and Pfister, 1981)). In the dorsal striatum of sham rats (A), the typical caudate-putamen cytoarchitecture was appreciable, with numerous transversally sectioned white matter *fascicula* (f) surrounded by gray matter containing diverse types of neurons, distinct on the bases of their size and shape (Danner and Pfister, 1981). In vehicle-treated rats, the striatal tissue was clearly damaged, the cytoarchitecture was lost, the *fascicula* were much less recognizable, the distinction between white and gray matter no more appreciable, the interstitial spaces increased, and numerous heterochromatic small nuclei were present (shown by the arrows in the insets, Figure 3B1). In the fronto-parietal cortex, the columnar organization was no longer appreciable, the interstitial spaces were enlarged and dilated, and numerous heterochromatic small nuclei were present (shown by the arrows in the insets, Figure 3E1). No heterochromatic nuclei were ever found in the striatum and cortex of sham rats. Qualitative analysis (Figures 3G,H) shows that administration of BAY60-6583 at the dose of 0.1 mg/kg, was associated with a recovery of staining intensity, and with a reduction of heterochromatic small nuclei in both brain regions. In BAY60-6583-treated rats, the cytoarchitecture of the white matter *fascicula* was maintained in the dorsal corpus striatum (Figure 3C) and the columnar organization was still appreciable in the fronto-parietal cortex (Figure 3F). Quantitative analysis showed that treatment with BAY60-6583 significantly reduced the number of heterochromatic nuclei in the ischemic striatum (−72.4%, *p < 0.02 vs. vehicle-treated rats, unpaired Student’s t test; Figure 3G) and in the ischemic cortex (−84.4%, **p < 0.005 vs. vehicle-treated rats, unpaired Student’s t test; Figure 3H).

### Effect of Treatment With the Adenosine A_2B_ Receptor Agonist on Neuronal Damage 7 days After tMCAo

**FIGURE 4:**
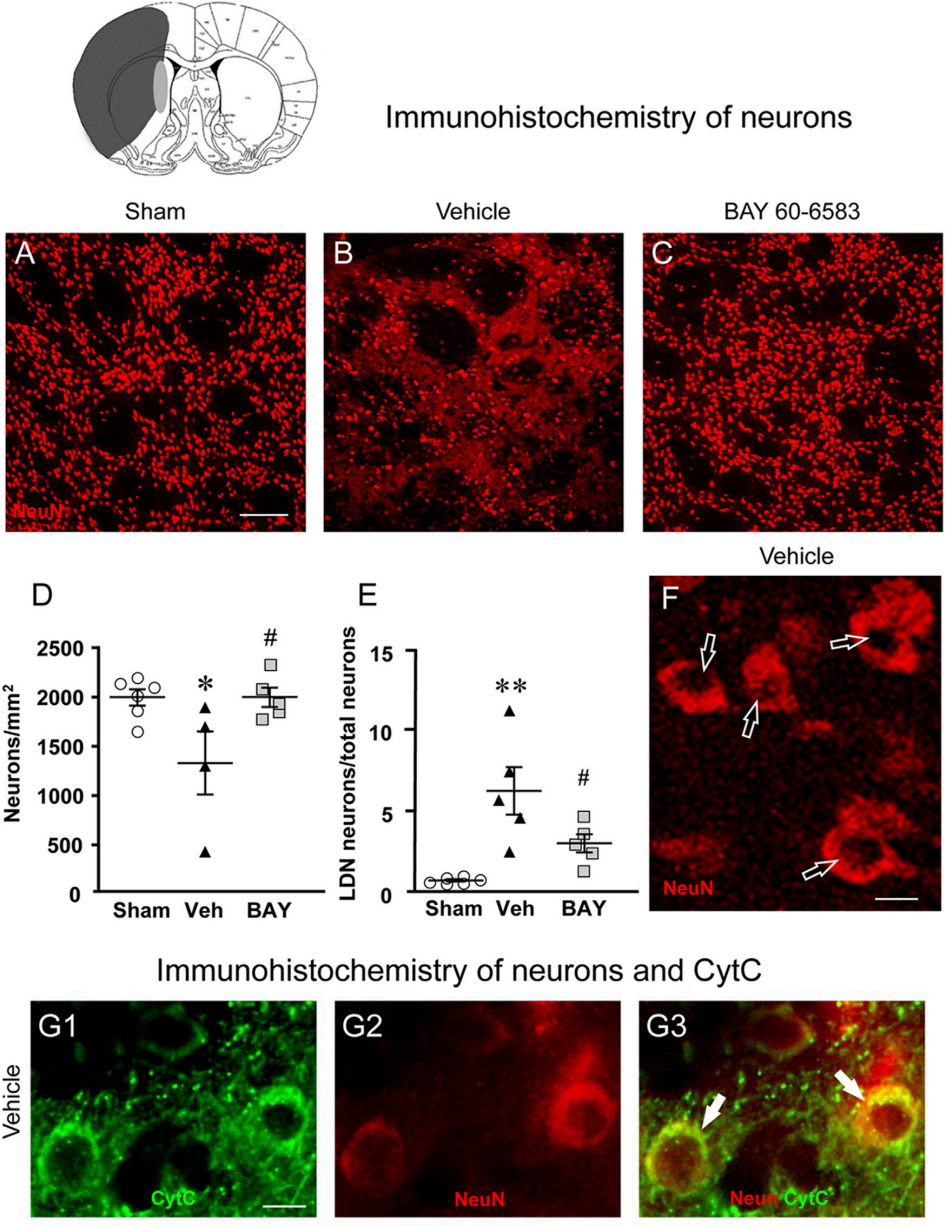
Analysis of neurons of sham, vehicle- and BAY60**-**6583-treated rats 7 days after tMCAo. Upper part: Schematic brain picture of a coronal section (at Bregma = 0, König et al., 1964) showing the infarct area in a vehicle-treated rat. The gray circle indicates region within the boundary zone where photomicrographs were captured. **(A–C)** Representative confocal microphotographs of NeuN immunostaining of neurons (red) in the ipsilateral striatum of a sham **(A)**, a vehicle- **(B)** and a BAY60-6583-treated **(C)** rat. Scale bar = 100 μm. **(D)** Quantitative analysis of the density of neurons (neurons/mm^2^) in the ROI of sham (n = 6), vehicle- (n = 4) and BAY60-6583-treated (n = 5) rats. Neuronal density was significantly lower in the striatum of tMCAo rats treated with vehicle in comparison to sham rats. BAY 60-6583 reverted significantly this effect. Significativity: *p < 0.05 vehicle-treated vs. sham rats; #*p* < 0.05 BAY60-6583- vs. vehicle-treated rats, One-way ANOVA followed by Newman-Keuls post hoc test. **(E)** Quantitative analysis of NeuN-positive LDN neurons in the striatum of sham (n = 6), vehicle- (n = 5) and BAY60-6583-treated (n = 5) rats, expressed as percent of total neurons in the ROI. The percent of LDN neurons was significantly higher in striatum of vehicle-treated vs. sham rats. BAY 60-6583 reverted significantly this increase. Significativity: **p < 0.01 vehicle-treated vs. sham rats; #*p* < 0.05 BAY60-6583- vs. vehicle-treated rats, One-way ANOVA followed by Newman-Keuls post hoc test. Data are reported as individual data points (mean ± SEM is also shown). **(F)** Note the presence of many LDN neurons (open arrows) in the striatum of vehicle-treated rats. Scale bar = 7.5 μm. **(G1-G3)** Representative microphotographs of CytC (**G1**, green), NeuN (**G2**, red) immunostaining of LDN neurons, and the merge of the previous images (**G3**, orange-yellow) in the ipsilateral striatum of a vehicle-treated rat. The arrows show the colocalization of CytC and NeuN immunostaining in the neuronal cytoplasm. Scale bar: 7.5 µm.

To estimate the damage caused by 1 h tMCAo to neurons and the effect of treatment with BAY60-6583 in the striatal boundary zone, we performed fluorescent confocal immunohistochemical analysis of neurons using the neuron specific anti-NeuN antibody in striatal sections from sham rats, from vehicle-treated rats and from BAY60-6583-treated rats. Representative confocal images (each obtained stacking 10 consecutive scans, z step of 1.2 μm, total thickness 12 μm) of NeuN immunostaining in the ipsilateral striatal boundary zone are shown in Figures 4A–C. Figure 4D shows the quantitative analysis of NeuN-positive cells in the striatum of sham, vehicle- and BAY60-6583 rats. Statistical analysis performed with One-way ANOVA showed that the density of NeuN positive neurons decreased significantly in vehicle-treated rats in comparison to sham rats (F_2,12_ = 4.788, *p* < 0.05; −34%, *p < 0.05, vehicle-treated vs. sham rats, Newman-Keuls post hoc test). The treatment with BAY60-6583 significantly antagonized this effect (^#^ *p* < 0.05, BAY60-6583- vs. vehicle-treated rats, Newman-Keuls post hoc test). Indeed, the density of neurons in BAY60-6583-treated rats was not different from that of sham rats (+0.01%, n.s., BAY60-6583-treated vs. sham rats). In the ROI of the contralateral striatum we found no difference in the density of neurons among the three experimental groups (data not shown).

Detailed analysis of NeuN immunostained neurons in the boundary zone of ipsilateral striatal sections was performed on confocal z projections (each obtained stacking three consecutive scans, z step 1.2 μm each, total thickness 3.6 μm) of NeuN immunostaining. A representative image of a vehicle-treated rat is shown in Figure 4F. Figure 4F shows that in vehicle-treated rats many of the surviving neurons were damaged, as shown by signs of karyorrhexis (Figure 4F). Indeed, we observed many neurons that had lost their nuclear NeuN positive immunofluorescence (Figure 4F, open arrows), which is a clear index of damaged nuclei, while NeuN positive immunofluorescence persisted in the cytoplasm. We had previously found and characterized neurons showing karyorrhexis in different models of ischemia *in vitro* (Fusco et al., 2018) and *in vivo* (Lana et al., 2017), and we had named them Low Density Nucleus neurons, “LDN neurons”. Here we quantified LDN neurons in the boundary zone of ipsilateral striatal sections of sham, vehicle- and BAY60-6583-treated rats (Figure 4E). Statistical analysis performed with One-way ANOVA showed that the percent of LDN neurons over total (surviving) neurons was significantly higher in vehicle-treated rats compared with sham rats (F_2,13_ = 10.85, *p* < 0.002; +780%, **p < 0.01 vehicle treated vs. sham rats, Newman-Keuls post hoc test). Interestingly, treatment with BAY60-6583 decreased significantly, although not completely, the percent increase of LDN neurons/total neurons in the ipsilateral striatum (−52%, ^#^ *p* < 0.05, BAY60-6583- vs. vehicle-treated rats, Newman-Keuls post hoc test). Indeed, the percent of LDN neurons in BAY60-6583-treated rats was not significantly different from that in sham rats (n.s., sham vs. BAY60-6583-treated rats).

To determine whether the above-described effects might be caused by apoptosis, we used double immunohistochemistry for NeuN and CytC, a protein which, in the most advanced stages of apoptosis, is intensely and diffusely released from mitochondria to the cytoplasm, where it activates caspases (Kluck et al., 1997; Yang et al., 1997; Jiang and Wang, 2004; Suen et al., 2008). CyTC can be used as a marker of apoptosis by immunohistochemistry (Martínez-Fábregas et al., 2014). The results are shown in Figures 4G1–G3 where it is possible to visualize that CytC (Figure 4G1, green) is colocalized in the cytoplasm of two LDN neurons (Figure 4G2, red), as evidenced in the merge of the previous pictures (Figure 4G3), indicating that LDN neurons are apoptotic.

### Effect of Treatment With the Adenosine A_2B_ Receptor Agonist on Total Microglia 7 days After tMCAo

**FIGURE 5:**
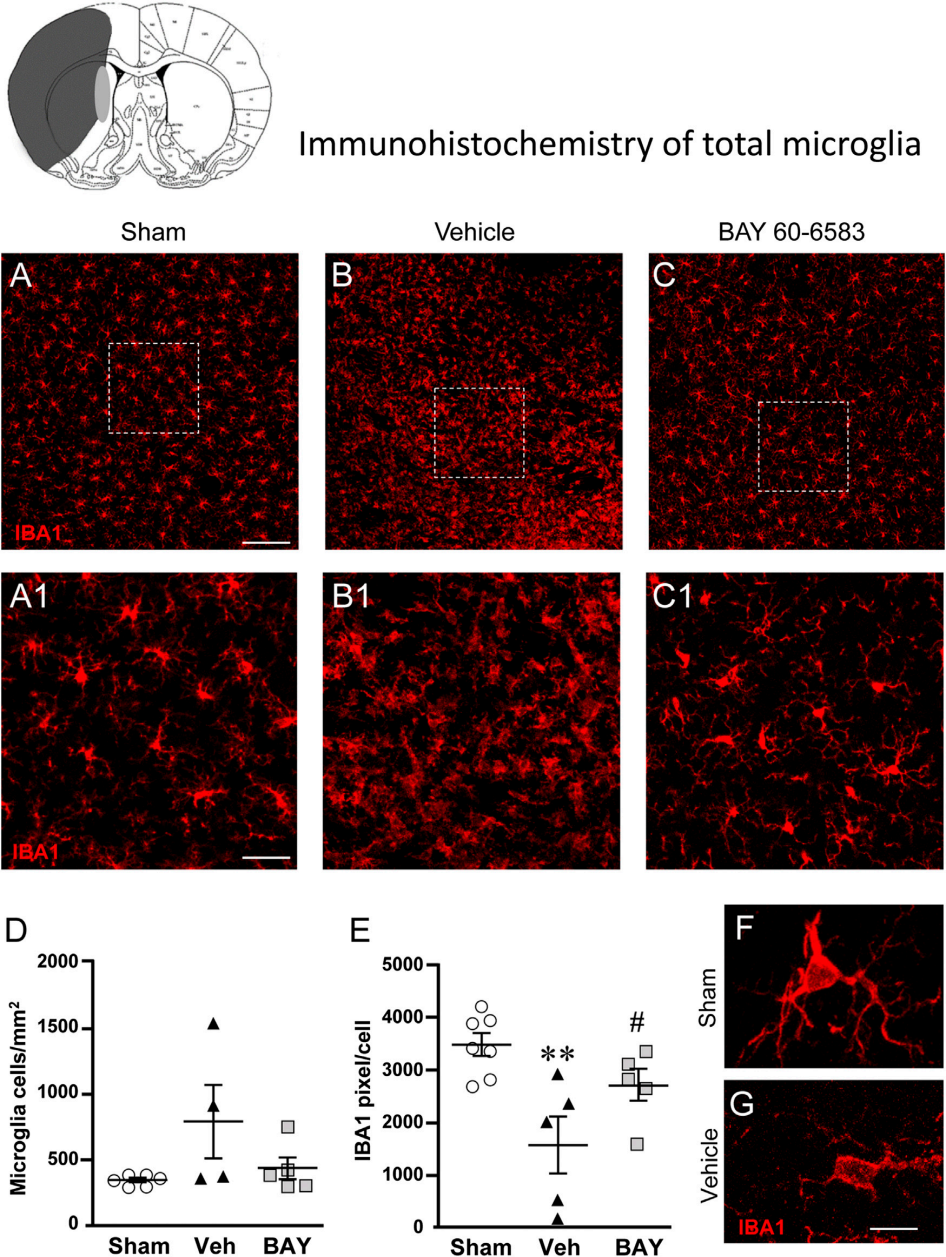
Analysis of microglia of sham, vehicle- and BAY60**-**6583-treated rats 7 days after tMCAo. Upper part: Schematic brain picture of a coronal section (at Bregma = 0) (König et al., 1964) showing the infarct area in a vehicle-treated rat. The gray circle indicates region within the boundary zone where photomicrographs were captured. **(A–C1)** Representative confocal photomicrographs of IBA1 immunostaining of microglia (red) in the striatum of a sham **(A)**, a vehicle- **(B)**, and a BAY60-6583-treated **(C)** rat. Scale bar = 100 μm. Panels **(A1–C1)** show the magnification of areas framed in Panels **(A–C)**. Scale bar = 20 μm. **(D)** Quantitative analysis of IBA1 positive microglia/mm^2^ in striatum of sham (n = 5), vehicle- (n = 5) and BAY60-6583-treated (n = 6) rats. **(E)** Quantitative analysis of IBA1 positive pixels/cell in the striatum of sham (n = 5), vehicle- (n = 5), and BAY60-6583-treated (n = 6) rats. Significativity: **p < 0.01 vehicle-treated vs. sham rats, ^#^ *p* < 0.05 BAY60-6583- vs. vehicle-treated rats, One-way ANOVA followed by Newman-Keuls post hoc test. Data are reported as individual data points (mean ± SEM is also shown). **(F,G)** Representative confocal z projections (z stacks of 65 consecutive scans, each 0.4 µm, total thickness 26 µm) of a microglia cell from a sham **(F)** and a vehicle-treated **(G)** rat. Scale bar: 5 µm.

To estimate the damage caused by 1 h tMCAo to microglial cells and the effect of treatment with BAY60-6583 in the striatal boundary zone, we performed fluorescent confocal immunohistochemical analyses of microglia in sections from sham rats, from vehicle-treated rats and from BAY60-6583-treated rats. Total microglia were identified using the fluorescent immunostaining for IBA1, as shown by the representative images in Figures 5A–C, each obtained stacking 10 consecutive confocal z scans (1.2 µm each, total thickness 12 µm) through the thickness of the 30 µm-thick striatal sections. Seven days after the ischemic insult, we found remarkable differences in the morphology of microglia in the ipsilateral striatum of vehicle-treated rats in comparison to sham rats (Figure 5B1). Figures 5A1–C1 show the magnifications of the framed areas in Figures 5A–C. In vehicle-treated rats (Figures 5B1,G), we found a strong pattern of microglia activation: microglia cells appeared round-shaped with shorter and less numerous branches (amoeboid-like) compared with sham rats (Figures 5A1,F) in which microglia showed highly ramified and more numerous branches that occupy a large spatial domain, typical of resting microglia. BAY60-6583 reduced the strong pattern of microglia activation, reverting these morphological changes (Figure 5C1). Indeed, the quantitative analysis of IBA1-positive cells in Figure 5D revealed that in vehicle-treated rats the density of microglia cells increased, although not significantly, (+130%, n.s., One-way ANOVA, vehicle-treated vs. sham rats), in comparison to sham rats. Analysis of IBA1 positive pixel/cell (Figure 5E) with One-way ANOVA (F_2,14_ = 7.675, *p* < 0.01) indicated that in vehicle-treated rats the number of pixel/cell was significantly decreased (−55%, **p < 0.01 vehicle-treated vs. sham rats, Newman-Keuls post hoc test). Since the volume of a microglial cell in ameboid state is reduced in comparison to a resting cell, the number of pixel/cell indirectly confirms the reactive state of the cell. Indeed, microglia cells appeared round-shaped, with shorter branches (Figure 5G) in comparison to sham rats (Figure 5F). Therefore, from the data presented in Figures 5D,E, it is possible to conclude that in vehicle-treated rats the number of microglia cells increased (although not significantly), and the cells were in an activated state. The density of microglia cells in BAY60-6583-treated rats was not different from that of sham rats (Figure 5D). Treatment with BAY60-6583 caused a significant increase in pixel/cell (^#^ *p* < 0.05, BAY60-6583-treated vs. vehicle-treated rats, One-way ANOVA followed by Newman-Keuls post hoc test) (Figure 5E) indicating that it has significantly protected microglia from reactivity (see Figure 5C1).

### Effect of Treatment With the Adenosine A_2B_ Receptor Agonist on Astrocytes Morphology 7 days After tMCAo

To estimate whether 1 h tMCAo modifies the density and reactivity of astrocytes and the effect of treatment with BAY60-6583, we performed fluorescent confocal immunohistochemical analyses with anti-GFAP antibody on sections of the striatal boundary zone from sham, vehicle- and BAY60-6583-treated rats.

**FIGURE 6:**
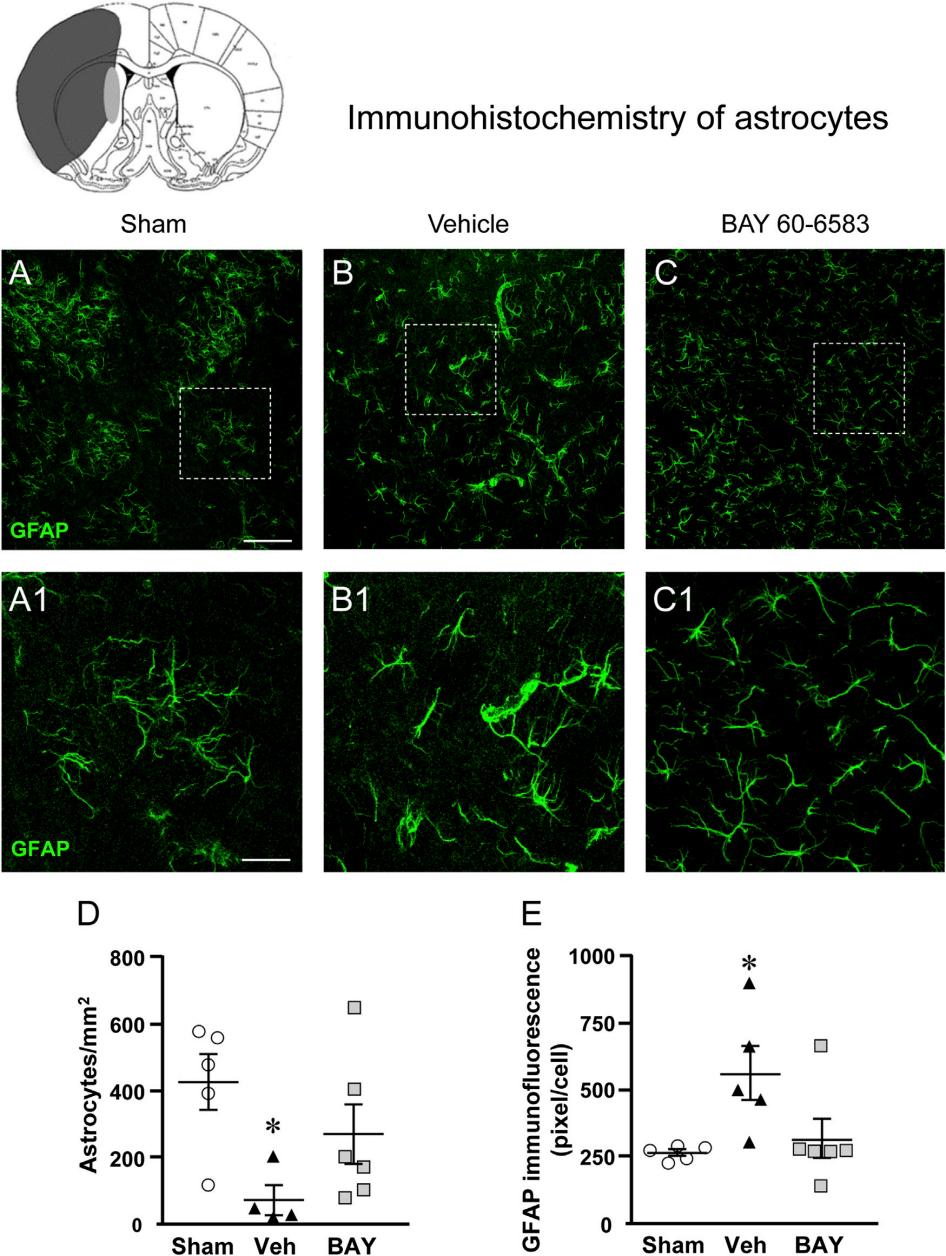
Analysis of astrocytes of sham, vehicle- and BAY60**-**6583-treated rats 7 days after tMCAo. Upper part: Schematic brain picture of a coronal section (at Bregma = 0) (König et al., 1964) showing the infarct area in a vehicle-treated rat. The gray circle indicates region within the boundary zone where photomicrographs were captured. **(A–C1)** Representative confocal microphotographs of GFAP immunostaining of astrocytes (green) in the striatum of a sham **(A)**, a vehicle- **(B)**, and a BAY60-6583-treated **(C)** rats. Scale bar = 100 μm. Panels **(A1–C1)** show the magnification of areas framed in Panels **(A–C)**, respectively. Scale bar = 30 μm. **(D)** Quantitative analysis of GFAP positive astrocytes/mm^2^ in the striatum of sham (n = 5), vehicle- (n = 5) and BAY60-6583- (n = 6) rats. Significativity: *p < 0.05 vehicle-treated vs. sham rats, One-way ANOVA followed by Newman-Keuls post hoc test. Astrocytes were significantly less numerous in striatum of vehicle-treated rats in comparison to sham rats. BAY60-6583 reverted this effect. **(E)** Quantitative analysis of GFAP in the striatum of sham (n = 5), vehicle- (n = 5), and BAY60-6583-treated (n = 6) rats. Significativity: *p < 0.05 vehicle-treated vs. sham rats and BAY60-6583-treated rats, One-way ANOVA followed by Newman-Keuls post hoc test. Data are reported as individual data points (mean ± SEM is also shown).

Figures 6A–C shows the representative confocal images (each obtained stacking 10 consecutive z scans, 1.2 µm each, total thickness 12 µm) of GFAP immunostaining. Panels 6A1-C1 show the magnifications of the framed areas in Figures 6A–C. Seven days after the ischemic insult, the density and morphology of astrocytes in the boundary zone of the ipsilateral striatum of vehicle-treated rats were significantly different from those of the sham rats (Figure 6B1). Treatment with BAY60-6583 appeared to revert the alterations of the density and morphology of astrocytes caused by ischemia (Figure 6C1). Quantitative analysis of GFAP positive astrocytes reported in Figure 6D, shows that in vehicle-treated rats the density of GFAP positive astrocytes decreased significantly in comparison to sham rats (One-way ANOVA: F_2,12_ = 4.137, *p* < 0.05; −83%, *p < 0.05 vehicle-treated vs. sham rats, Newman-Keuls post hoc test, Figure 6A1). Treatment with BAY60-6583 tended to revert this effect (−28%, n.s., BAY60-6583 vs. sham). Although astrocytes of vehicle-treated rats were decreased in number, the number of GFAP positive pixel/cell (Figure 6E) was significantly higher than in sham rats and BAY60-6583 reverted significantly this effect (One-way ANOVA: F_2,13_ = 4.547, *p* < 0.05; +107%, *p < 0.05, vehicle-treated vs. sham rats and BAY60-6583-treated rats, Newman-Keuls post hoc test; +19%, n.s., BAY60-6583-treated vs. sham rats).

Since the volume of reactive, hypertrophic, astrocytes is increased in comparison to resting astrocytes, the increase of the number of pixel/cell in vehicle-treated rats indirectly confirms that astrocytes are more activated than those of the sham rats and that BAY60-6583 has significantly protected astrocytes from reactivity (see Figure 6C1).

### Effect of Treatment With the Adenosine A_2B_ Receptor Agonist on Cytokine Plasma Levels After tMCAo

**FIGURE 7:**
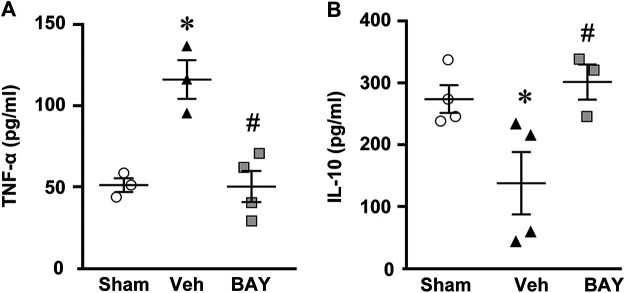
Effect of chronic treatment with BAY60-6583 (0.1 mg/kg i.p.) on TNF-α **(A)** and IL-10 **(B)** plasma levels. Results are expressed as pg of protein/ml of plasma in sham (n = 3, 4), vehicle- (n = 3, 4) and BAY60-6583-treated (n = 3, 4) rats. Values are mean ± SEM and individual data points are shown: *p < 0.05, vehicle-treated vs. sham rats; ^#^ *p* < 0.05 BAY60-6583- vs. vehicle-treated rats, One-way ANOVA followed by Newman-Keuls post hoc test.

Seven days after tMCAo, plasma levels of the pro-inflammatory cytokine TNF-α were significantly higher in vehicle-treated rats than in sham rats (One-way ANOVA: F_2,7_ = 17.51, *p* < 0.001; *p < 0.05, vehicle-treated vs. sham rats, Newman-Keuls post hoc test; Figure 7A). The plasma levels of IL-10, a regulatory anti-inflammatory cytokine, were significantly lower in vehicle-treated rats than in sham rats (One-way ANOVA: F_2,8_ = 7.03, *p* < 0.01; *p < 0.05, vehicle-treated vs. sham rats, Newman-Keuls post hoc test; Figure 7B). Treatment with BAY60-6583 significantly decreased TNF-α levels (^*^ *p* < 0.05, BAY60-6583 vs. vehicle-treated rats, Newman-Keuls post hoc test; Figure 7A) and significantly increased IL-10 levels (^*^ *p* < 0.05, BAY60-6583 vs. vehicle-treated rats, Newman-Keuls post hoc test; Figure 7B) to those of sham rats.

### Effect of Treatment With the Adenosine A_2B_ Receptor Agonist on Granulocytes Infiltration After tMCAo

**FIGURE 8:**
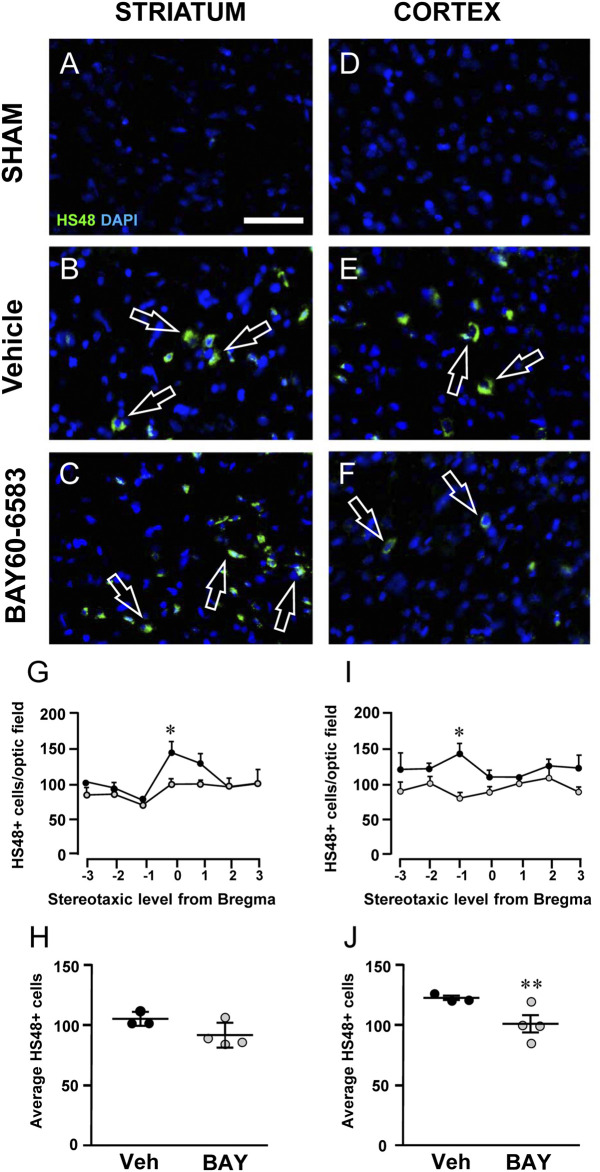
Effect of chronic treatment with BAY60-6583 (0.1 mg/kg i.p.) on blood cell infiltration in the ischemic core 2 days after tMCAo. **(A–F)** Representative microphotographs of HIS-48 positive cells (green label, open arrows) detected in ischemic striatum **(A–C)** and cortex **(D–F)** of sham, vehicle- and BAY60-6583-treated rats taken at AP = 0 from Bregma (König and Klippel, 1967). Cell nuclei were labeled with DAPI (blue). Scale bar = 50 µm. **(G–J)** Quantitative analyses of HIS-48^+^ cells in the striatum **(G,H)** and cortex **(I,J)** of vehicle- and BAY60-65803-treated rats. **(G,I)** Number of HIS-48 positive cells per optical field counted in seven coronal levels of striatum **(G)** and cortex **(I)** between AP = +2.0 mm to −1.0 mm from Bregma of vehicle- (n = 3) and BAY60-6583-treated rats (n = 4). Significativity was evaluated by two-way ANOVA followed by Bonferroni post hoc test (*p < 0.05 vs. vehicle-treated rats). **(H,J)** Average of HIS-48 positive cells between AP = +2.0 mm to −1.0 mm from Bregma of vehicle- (n = 3) and BAY60-6583-treated rats (n = 4). Significativity was evaluated by unpaired Student’s t-test (**p < 0.01 vs. vehicle-treated rats). Data in the graphs are mean ± SEM and individual data points are shown.

Granulocytes were evaluated using anti-HIS-48 antibody, as shown in Figures 8A–F. Infiltration of HIS-48 positive granulocytes was never found in any section of the striatum and cortex of sham rats (n = 3). On the contrary, in both cortical and striatal ischemic areas of vehicle-treated rats, HIS-48 positive cells were clearly detectable 2 days after tMCAo (Figures 8B–F, open arrows). BAY60–6583 reduced significantly the number of HIS48 positive cells in the cortical and striatal ischemic core 2 days after tMCAo. In the cortex, the effect was significant at Stereotaxic level −1 from Bregma (*p < 0.05, two-way ANOVA followed by Bonferroni post hoc test, Figure 8I). Statistical analysis performed with two-way ANOVA, calculated for the two factors: Treatment and Stereotaxic levels, showed that Treatment (F_1,35_ = 18.82; *p* < 0.0001) was statistically significant, while Stereotaxic levels (F_6,35_ = 0.4687; *p* < 0.8) and the Interaction between Treatment and Stereotaxic levels (F_6,35_ = 1.057; *p* < 0.4) were not significant. In the striatum, treatment with BAY60-6583 significantly reduced the number of HIS48 positive cells at Stereotaxic level 0 (*p < 0.05, two-way ANOVA followed by Bonferroni post hoc test, Figure 8G). Two-way ANOVA, calculated for the two factors: Treatment and Stereotaxic levels, showed that Treatment (F_1,28_ = 6.846; *p* < 0.01) and Stereotaxic levels (F_6,28_ = 5.322; *p* < 0.0009) were statistically significant, but the Interaction between Treatment and Stereotaxic levels (F_6,28_ = 1.485; *p* < 0.2) was not. The mean effect of BAY60-6583 was significant between −3 and +3 stereotaxic levels in the cortex (**p < 0.004, unpaired Student’s t test, Figure 8J), but the effect did not reach statistical significance in the ischemic striatum (n.s., unpaired Student’s t test, Figure 8H). Seven days after tMCAo, the infiltration of granulocytes was no longer detectable in the ischemic tissues (Melani et al., 2014).

## Discussion

Our results demonstrate that the selective adenosine A_2B_ receptor agonist BAY60-6583, administered chronically and systemically at the dose of 0.1 mg/kg, protected from the neurological deficit up to 7 days after tMCAo. The second day after tMCAo BAY60-6583 reduced blood cell infiltration, and 1 week after tMCAo BAY60-6583 significantly reduced the infarct volume and the ischemic brain damage, counteracted neuron degeneration, microglia activation and protected astrocytes from death.

Vehicle-treated rats after ischemia showed a functional deficit of moderate gravity, which spontaneously tended to recover up to 7 days after the insult. Treatment with BAY60-6583 significantly reduced the neurological deficit from 24 h after ischemia, indicating that it improved significantly the functional recovery, at least up to 7 days after ischemia.

The ischemic rats had a significant loss of body weight, which was not restored to normal by the treatment with BAY60-6583. In this regard, it is worth mentioning that adenosine released from the adipose tissue (Schwabe et al., 1975; Capogrossi et al., 1986) participates in the regulation of adipocyte function (Eisenstein and Ravid, 2014). In particular, stimulation of A_2B_ receptors inhibits adipogenesis (Gharibi et al., 2012). The effects of A_2B_ receptor agonists can help explaining the lack of protection of BAY60-6583 from the body weight loss.

Seven days after ischemia, a definite infarct area is still detectable, and the triple labeling immunohistochemistry revealed remarkable qualitative and quantitative alterations of neurons, astrocytes and microglia in the striatum of tMCAo vehicle-treated rats in comparison to sham rats.

Seven days after ischemia, analysis of astrocytes in vehicle-treated rats showed that their density was significantly decreased and that they were more activated in comparison to sham rats. Astrocytes showed morphological modifications that characterize clasmatodendrosis such as cytoplasmic swelling, vacuolization of the astrocyte soma, beading and fragmentation of their branches (Qin et al., 2010). Clasmatodendrotic alterations in cell morphology are directly related to changes in cell function (Hulse et al., 2001; Hinson et al., 2007) and an association between astrocyte injury and disruption of gliovascular interactions of the BBB has been described in elderly post-stroke survivor patients (Chen et al., 2016). Our results demonstrated that the selective A_2B_ receptor agonist BAY60-6583 had protective effects on the ischemia-induced loss of astrocytes and their activation in the striatum. In isolated astrocytes, the A_2B_ receptor activates phospholipase C (PLC) (Pilitsis and Kimelberg, 1998) and is responsible for the adenosine-induced stimulation of Interleukine-6 (IL-6) (Schwaninger et al., 2002). In the *in vitro* model of hypoxia/reoxygenation A_2B_ receptor stimulation increases IL-6 from rat astrocytes cultures (Maeda et al., 1994) and IL-6 exerts neuroprotective actions in a rat model of permanent MCAo (Loddick et al., 1998). Furthermore, activation of A_2B_ receptor on murine astrocytes during glutamate-induced neuronal stress (Moidunny et al., 2012), as well as in an *in vivo* model of cortical brain injury (Banner et al., 1997) and after cerebral ischemia in the rat (Suzuki et al., 2000), is responsible for increased production of leukemia inhibitory factor (LIF), a member of the IL-6 family. Indeed, it has been shown that LIF, when up-regulated in astrocytes and neurons after A_2B_ receptor activation, is related to anti-inflammatory processes and cell protection (Rosi et al., 2003; Yang et al., 2006; Kuno et al., 2007).

Seven days after ischemia, a pattern of strong IBA-1 positive cells activation was evident. Seven days after ischemia the labeling by IBA-1 is likely attributable to microglia cells because although both microglia and macrophages greatly increase in the ischemic hemisphere starting from about 24 h after MCAo, remain at maximal levels around 3–4 days thereafter (Gaire et al., 2015), than while macrophages slowly decrease microglia is still high 7 days thereafter (Gelderblom et al., 2009). The microglia activation was detected in the striatal boundary zone of tMCAo vehicle-treated rats where microglia cells not only were more numerous but also appeared in a reactive, ameboid state in comparison to sham rats. Treatment with BAY60-6583 decreased microglia cell density and rescued the morphology of microglia cells to a resting state. A_2B_ receptors are expressed on microglia cells (Hammarberg et al., 2003; Van Calker and Biber, 2005; Daré et al., 2007) and their activation reduces the expression of TNF-α in primary microglia cultures (Merighi et al., 2015) and increases IL-10 production from murine microglial cells (Koscsó et al., 2012) with consequent anti-inflammatory effects. It is well known that after an ischemic event, over-activation of microglia is detrimental and that these resident immune cells are involved in the neuroinflammation process (Schwartz, 2003; Kriz, 2006; Block et al., 2007). It is therefore plausible that the effect of BAY60-6583 on A_2B_ receptor located on microglia is responsible for a decreased release of TNF-α and increased release of IL-10, with resulting protective effects of rescuing the resting state of microglia.

In accordance with the above results, seven days after ischemia, H&E staining showed that chronic treatment with BAY60-6583 reconstituted the cytoarchitecture in cortex and striatum and decreased heterochromatic small nuclei that belong to astrocytes and microglia (Melani et al., 2014).

Overall, the previous observations indicate that a protective effect of chronic BAY60-6583 treatment up to 7 days after ischemia can be attributed to direct agonism of A_2B_ receptor located on rat microglial and/or astrocytic cells.

In the striatal boundary zone of tMCAo vehicle-treated rats, we found remarkable signs of neurodegeneration 7 days after ischemia. Indeed, we found significant increase of neuronal death, evidenced by loss of neurons, and most of the surviving neurons had evident signs of damage. Indeed, immunohistochemistry showed that surviving striatal neurons in the boundary zone had signs of karyorrhexis and possibly nuclear fragmentation, as evidenced by the significantly higher number of Low Density Nucleus (LDN) neurons. As previously demonstrated (Cerbai et al., 2012; Lana et al., 2016; Lana et al., 2017; Fusco et al., 2018), here we confirmed that LDN neurons are highly positive for CytC, which demonstrates that they are apoptotic. Indeed, nuclear fragmentation is one of the characteristics of apoptosis and in all our previous papers we had demonstrated that LDN neurons are in late phase of apoptosis (Cerbai et al., 2012; Fusco et al., 2018; Lana et al., 2016; Lana et al., 2017). The interesting data of our present manuscript is that in BAY60-6583-treated rats we found a significant decrease of both cell death and of damaged, LDN neurons. Indeed, BAY60-6583 rescued the neuronal loss and reduced significantly the percent of LDN neurons on total neurons, thus demonstrating its protective role against neurodegeneration.

Adenosine A_2B_ receptors in the brain are expressed on neurons (Liu et al., 2019) and in the mouse hippocampus on the glutamatergic terminals where their selective stimulation counteracts the predominant adenosine A_1_ receptor-mediated inhibition of synaptic transmission (Gonçalves et al., 2015). In agreement, in the acute *in vitro* model of OGD in rat hippocampal slices, neuronal A_2B_ receptors are involved in promoting brain excitotoxicity and neuronal damage in the CA1 area (Fusco et al., 2018). These results seem to be in contrast with the results of the present paper where the adenosine A_2B_ agonist BAY60-6583 proves protective against neuronal ischemic damage. However, it must be taken into account that after ischemia, the damage to the tissue is the result of a combination of the precocious acute excitotoxic damage followed by a secondary neuroinflammatory damage that develops from hours to days after the ischemic insult and that is generated by the interplay of resident glial cells with peripheral infiltrating blood cells (Dirnagl et al., 1999). Days following ischemia, the early harmful role of A_2B_ receptors located on neurons appears overcome by activation of A_2B_ receptors located on glial cells and on peripheral blood cells. Indeed, besides glial and neuronal cells, A_2B_ receptors are also present on endothelial (Feoktistov et al., 2004) and hematopoietic cells (Gessi et al., 2005; Eckle et al., 2008). Protection exerted by intravenous treatment with BAY60-6583, within 24 h after ischemia, has been attributed to protection of the endothelium due to stimulation of the tissue inhibitor of matrix metalloproteinases-1 (TIMP-1), to inhibition of tPA-induced matrix metalloprotease (MMP) activation, and prevention of tight junction protein degradation. (Li et al., 2017). Interestingly, in the presence of tPA (administered after ischemic stroke as thrombolytic agent), BAY60-6583 reduces tPA-induced hemorrhages 24 h after ischemia (Li et al., 2017).

After tMCAo and subsequent reperfusion, an initial increase of BBB permeability (Sandoval and Witt, 2008) is followed by a biphasic increase at 5 and 72 h (Kuroiwa et al., 1985). Changes in BBB are responsible for cell infiltration. Infiltrated neutrophils expressing cytokines and chemotactic factors promote expansion of the inflammatory response in the ischemic tissue (Haskó and Pacher, 2008). Indeed, correlations among neutrophil accumulation, severity of brain tissue damage and neurological outcome have been reported (Akopov et al., 1996). Our observation that 2 days after tMCAo, BAY60-6583 significantly reduced granulocyte infiltration in the cortex supports the idea that A_2B_ receptor activation on endothelial and blood cells is involved in counteracting neutrophils infiltration and then inflammation of brain parenchyma. In agreement, it has been demonstrated that A_2B_ receptor KO mice, exposed to hypoxia, exhibit increased neutrophils infiltration in the brain (Eckle et al., 2007; Eckle et al., 2008). Importantly, the observation that BAY60-6583 proved protective from granulocyte infiltration in the cortex 48 h after ischemia and from the functional deficit as early as 24 h after ischemia, indicates that A_2B_ receptor agonists can be an important therapeutic strategy to control brain injury progression since the first hours after ischemia.

Interestingly, chronic administration of BAY60-6583 antagonized both the increase of TNF-α and the decrease of IL-10 detected in plasma of vehicle-treated rats 7 days after ischemia. Such effects may be secondary to a central effect of BAY60-6583 that modifies pro-inflammatory cytokine production in the brain and consequently in peripheral blood levels. It is also possible that cytokine modifications in plasma reflects a direct peripheral mechanism of action of BAY60-6583 that reduces neutrophils activation. This possibility is supported by the evidence that adenosine A_2B_ receptor KO mice show increased basal levels of TNF-α and expression of adhesion molecules in lymphoid cells, resulting in increased leukocyte rolling and adhesion (Yang et al., 2006). Increasing evidences indicate a role for A_2B_ receptor in the modulation of inflammation and immune responses in selected pathologies like cancer, diabetes, as well renal, lung and vascular diseases (Borea et al., 2018). Indeed, stroke and inflammation are strictly interrelated. Brain ischemia induces profound inflammatory changes in peripheral organs (especially lungs and gut) as early as 2 h after tMCAo in mice as detected by whole body SPECT-based imaging protocols (Szigeti et al., 2015). Such peripheral inflammatory changes, in turn, may contribute to poorer recovery after stroke (Szigeti et al., 2015). The precise cellular-molecular mechanisms underlying these events are unclear, but, likely, they reflect a vicious circle responsible for changes of endothelial cells and of BBB permeability bringing to blood cell infiltration and further brain damage (the secondary damage after stroke). Overall, our results stress the key research questions of the predictive value of blood biomarkers in stroke and suggest that BAY60-6583, by controlling a secondary inflammatory damage, represents a new interesting pharmacological tool after brain ischemia.

The present study points toward the possibility that stimulation of A_2B_ receptors located on glial cells, on vascular endothelial cells and on blood cells attenuate the neuroinflammation that develops after ischemia. Importantly, the A_2B_ receptor agonists can be proposed as adjuvant therapy to the accepted pharmacological strategy with tPA and can be a promising strategy for decreasing the risk of hemorrhages during the treatment for ischemic stroke.

## Data Availability

The raw data supporting the conclusions of this article will be made available by the authors, without undue reservation.
